# Temporal Acoustic-Window Failure in Female Patients with Rheumatoid Arthritis: An Eight-Year Longitudinal Follow-Up Study

**DOI:** 10.3390/jcm15145654

**Published:** 2026-07-19

**Authors:** Attila Sas, Dávid Jónyer, Attila Valikovics, László Kostyál, Zsuzsanna Oláh, Katalin Hodosi, Zsófia Kardos, Csaba Oláh, Zoltán Szekanecz

**Affiliations:** 1 Doctoral School of Clinical Medicine, University of Debrecen, 4032 Debrecen, Hungary; 2 Department of Stroke-Neurology-Toxicology, Borsod-Abaúj-Zemplén County Central Hospital and University Teaching Hospital, 3526 Miskolc, Hungary; david.jonyer@gmail.com (D.J.); valikovics.idegtox@bazmkorhaz.hu (A.V.); 3 Faculty of Health Sciences, University of Miskolc, 3515 Miskolc, Hungary; dr.kardos.zsofia@gmail.com; 4 Department of Radiology, Borsod-Abaúj-Zemplén County Central Hospital and University Teaching Hospital, 3526 Miskolc, Hungary; kostyalfed@gmail.com; 5 Doctoral School of Health Sciences, University of Debrecen, 4032 Debrecen, Hungary; olahzsuzso@gmail.com; 6 Institute of Nursing Science and Diagnostic Imaging, University of Miskolc, 3515 Miskolc, Hungary; 7 Department of Internal Medicine, Faculty of Medicine, University of Debrecen, 4032 Debrecen, Hungary; hodosi@med.unideb.hu; 8 Department of Rheumatology, Borsod-Abaúj-Zemplén County Central Hospital and University Teaching Hospital, 3526 Miskolc, Hungary; 9 Department of Neurosurgery, Borsod-Abaúj-Zemplén County Central Hospital and University Teaching Hospital, 3526 Miskolc, Hungary; olahcs@gmail.com; 10 Mathias Institute, University of Tokaj, 3950 Sarospatak, Hungary; 11 Department of Rheumatology, Faculty of Medicine, University of Debrecen, 4032 Debrecen, Hungary; szekanecz.zoltan@med.unideb.hu

**Keywords:** rheumatoid arthritis, transcranial Doppler, transcranial color-coded duplex sonography, temporal acoustic window, temporal acoustic window failure, acoustic-window feasibility

## Abstract

**Background/Objectives:** Rheumatoid arthritis (RA) is associated with increased cerebrovascular risk and altered bone metabolism. Transcranial Doppler (TCD) and transcranial color-coded duplex sonography (TCCD) require an adequate transtemporal acoustic window (TAW), which may be limited by temporal bone thickness and structure. Our previous cross-sectional work showed a high frequency of temporal acoustic window failure (TAWF) in female RA patients. The present exploratory follow-up study evaluated whether binary TAW status showed detectable net progression over an 8-year interval. **Methods:** In 2017, 62 female RA patients and 60 age-matched female controls underwent standardized TCD/TCCD assessment and clinical/laboratory characterization. In 2023, 43 of the original RA patients were re-evaluated by the same experienced neurosonographer using standardized transtemporal insonation and TCCD verification when needed. TAW status was recorded bilaterally as existing TAW or TAWF and analyzed using paired transition counts and exact McNemar tests; treatment subgroup comparisons were exploratory. **Results:** Right-sided TAW was present in 27/43 patients and absent in 16/43 at both time points (TAWF prevalence 37.2%, 95% CI 24.4–52.1%). No right-sided discordant transition was observed (0/43; exact upper 95% bound 8.2%; exact McNemar *p* = 1.000). Left-sided TAW was present in 20/43 patients and absent in 23/43 at both time points (TAWF prevalence 53.5%, 95% CI 38.9–67.5%). Two left-sided discordant transitions were observed: one apparent gain and one apparent loss of TAW (2/43, 4.7%; exact 95% CI 0.6–15.8%; exact McNemar *p* = 1.000). Bilateral TAWF remained present in 15/43 patients at both time points. No statistically detectable differences in TAW status were observed among MTX, IFX, and TCZ groups, but these subgroup analyses were underpowered. **Conclusions:** In this treated female RA cohort, binary TAW status showed no detectable net progression over 8 years. These data do not prove true biological stability of skull sonographic permeability, and smaller changes cannot be excluded. Mechanistic links with disease control, vitamin D status, treatment exposure, or bone remodeling require larger studies with direct temporal bone imaging and semi-quantitative acoustic-window grading.

## 1. Introduction

Rheumatoid arthritis (RA) is a chronic immune-mediated inflammatory disease with important systemic and extra-articular consequences. In addition to joint damage, persistent inflammation, autoantibody formation, endothelial dysfunction, altered lipid metabolism and traditional cardiovascular risk factors contribute to accelerated atherosclerosis and increased cerebrovascular risk in RA [1,2,3,4,5]. Modern treat-to-target strategies, early conventional synthetic disease-modifying antirheumatic drugs (DMARDs), and escalation to biological or targeted synthetic therapies aim to reduce disease activity and long-term systemic complications [6,7].

Bone involvement is also central to RA. Marginal erosions, peri-articular osteopenia, generalized bone loss and fracture risk reflect an imbalance between osteoclast-mediated bone resorption and osteoblast-mediated bone formation. Pro-inflammatory cytokines, autoantibodies, glucocorticoid exposure, immobilization, vitamin D status and the RANK/RANKL/OPG axis link immune activation to altered bone remodeling [8,9,10]. Although cranial bone is not routinely assessed in RA, disease-related skeletal changes provide a plausible background for studying temporal bone ultrasound permeability [11].

TCD and TCCD provide repeatable bedside assessment of intracranial hemodynamics. Their use includes evaluation of major intracranial stenosis or occlusion, collateral flow, vasospasm, cerebral vasomotor reactivity, microembolic signals, right-to-left shunts and selected monitoring indications [11,12,13,14,15,16,17,18,19,20,21,22]. In RA, such methods are relevant because cerebrovascular injury may involve both macrovascular atherosclerosis and microvascular or endothelial dysfunction [23]. However, these applications require a usable cranial acoustic route to the basal cerebral arteries.

The transtemporal acoustic window is the most commonly used route for insonating the MCA, anterior cerebral artery, posterior cerebral artery and terminal internal carotid artery. TAWF may prevent or substantially limit TCD/TCCD assessment, especially when it is bilateral. The probability and quality of TAW are influenced by age, sex, ethnicity, temporal bone thickness, temporal bone density, diploic and cortical structure, pneumatization and soft-tissue characteristics [24,25,26,27,28,29,30,31,32,33,34,35]. Therefore, TAW status is not only a technical detail but also a determinant of whether longitudinal neurosonological follow-up is feasible.

From a physical perspective, transcranial ultrasound is possible because low-frequency ultrasound can penetrate selected thin skull regions. The temporal squama provides the best compromise between access and attenuation; however, small differences in thickness, density, diploic architecture, cortical layering, or overlying soft tissue may reduce signal intensity. TAWF is frequently recorded as a binary observation, but acoustic-window quality is better understood as a continuum ranging from excellent bilateral insonation to partial, poor-quality, or completely absent windows [28,29,30,31,32,33,34,35].

In our previous cross-sectional study, TAWF was substantially more common in female RA patients than in age-matched female controls and was associated with thicker and structurally heterogeneous temporal bones. TAWF was detected unilaterally or bilaterally in 34–53% of RA patients, compared with 13–20% of controls. Heterogeneous temporal bone structure was observed in 53–60% of RA patients but in only one control subject (3%). These findings suggested that altered bone structure in RA may reduce skull sonographic permeability [36].

The longitudinal behavior of TAW status in RA remains uncertain. It is not known whether TAWF progresses with aging and cumulative disease duration, remains largely unchanged once present, or can be modified by systemic disease control and bone metabolism-related factors. This uncertainty is clinically relevant because an initially adequate window could permit repeated MCA hemodynamic or cerebrovascular reactivity measurements, whereas bilateral TAWF may require alternative imaging strategies. The primary objective of this follow-up investigation was to describe paired changes in binary TAW status over an 8-year interval in female RA patients. Secondary objectives were to compare TAW status across MTX, IFX and TCZ treatment subgroups in an exploratory manner and to interpret the findings in the context of RA bone remodeling, TCD/TCCD feasibility, and the limitations of binary acoustic-window classification.

## 2. Materials and Methods

### 2.1. Study Design and Participants

**Figure 1:**
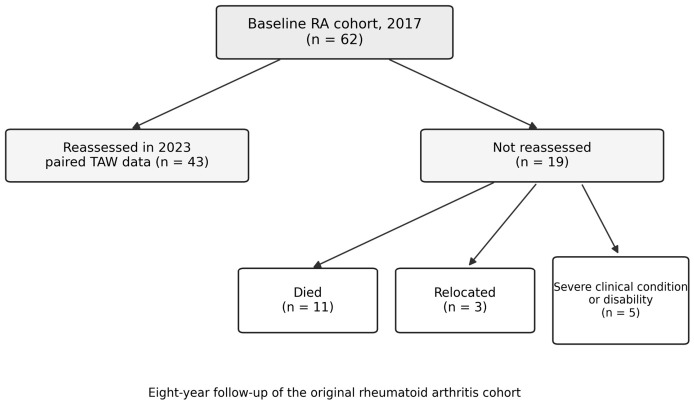
Patient flow from baseline to the 8-year follow-up. Of the 62 RA patients examined in 2017, 43 were reassessed in 2023 and had paired TAW data. Nineteen participants were not reassessed: eleven died, three relocated, and five were unable to participate because of severe clinical condition or disability.

This study represents a longitudinal follow-up of our 2017 RA-control neurosonological cohort. In 2017, 62 female patients fulfilling classification criteria for RA and 60 age-matched female controls were examined. In 2023, 43 of the original 62 RA patients were re-evaluated; 11 had died, 3 had relocated, and 5 were unable to undergo examination because of severe clinical condition or disability. The present analysis focuses on the 43 RA patients with paired TAW data. The 19 participants who were not reassessed are summarized in Figure 1. The healthy control group examined at baseline was not prospectively followed because the present study was designed as a longitudinal reassessment of the original RA cohort. Patients were enrolled after providing written informed consent following a detailed explanation of the research in accordance with the Declaration of Helsinki. The study was approved by the Regional Scientific and Research Ethics Committee (approval number: BORS-04/2023).

### 2.2. Neurosonological Protocol and Definition of Temporal Acoustic-Window Status

TCD/TCCD examinations were performed according to internationally accepted TCD practice standards and the ACR-AIUM-SPR-SRU practice parameter for transcranial Doppler ultrasound [13,22]. The same experienced neurosonographer (D.J.) performed the baseline and follow-up examinations. The same DWL Multi-Dop T digital TCD system (DWL Compumedics GmbH, Singen, Germany) was used, equipped with a 2 MHz pulsed-wave monitoring probe and a DiaMon recorder. Patients remained in the supine position and were examined while awake and calm. Adult transcranial insonation was performed through the transtemporal window located at the thinnest portion of the temporal bone, cephalad to the zygomatic arch and anterior to the ear, using a low-frequency probe suitable for skull penetration. Bilateral transtemporal insonation was attempted from standard anterior, middle and posterior temporal positions. The MCA was identified using the accepted transtemporal approach, spectral waveform, flow direction and depth criteria; normal MCA flow is directed toward the transducer, and the M1-MCA was searched and optimized within the standard 40–65 mm depth range, with the mid-M1 signal typically obtained at approximately 50–55 mm. Depth, gain, sample volume and probe angle were adjusted to obtain the strongest reproducible signal. Conventional spectral TCD was the initial examination method in all participants. TCCD was not routinely performed as a parallel test in every participant; it was used specifically as a verification step when the spectral TCD examination suggested absence of an acoustic window. For this verification, a B-mode-capable TCCD device (GE VIVID S5, GE Healthcare, Wauwatosa, WI, USA) with a 3ScRS 2 MHz probe was used. TCCD verification relied on B-mode landmarks and color flow mapping to reduce false-negative classification of insonation failure [22,28]. No standardized TCD/TCCD image archive was available at either time point for retrospective semi-quantitative grading. Follow-up examinations were not formally blinded to the baseline TAW classification.

TAW status was classified as ‘existing TAW’ when basal cerebral arteries, primarily the MCA, could be consistently insonated through the temporal region with reproducible signal quality sufficient for vessel identification. TAWF was recorded when adequate transtemporal insonation could not be achieved despite systematic bilateral search, standardized probe positioning, optimization of technical settings and repeated attempts, and when TCCD verification also failed to identify a suitable transtemporal signal at the expected arterial locations. The primary longitudinal endpoint was side-specific: right and left TAW status were classified and analyzed independently. Patient-level categories (bilateral existing TAW, unilateral TAW, and bilateral TAWF) were used only as descriptive summaries. Because acoustic-window quality is a continuum, the binary existing TAW/TAWF classification was used only because it was the consistently available variable at both time points. Formal semi-quantitative grading of window quality was not available.

### 2.3. Clinical and Laboratory Variables

Demographic characteristics, cardiovascular risk factors, RA disease features and treatment status were recorded. Disease activity was assessed using the 28-joint Disease Activity Score (DAS28). Laboratory parameters included inflammatory markers, lipid parameters, calcium-phosphate metabolism markers, vitamin D, osteocalcin and beta-C-terminal telopeptide of type I collagen. Laboratory analyses were performed using the available paired measurements for each analyte, and no imputation of missing laboratory values was performed. Treatment subgroup membership remained unchanged during follow-up; no patient switched between the MTX, IFX and TCZ groups. Medication adherence was not assessed using a validated adherence instrument or systematic pharmacy-refill persistence metrics. Vitamin D supplementation was clinician-directed as part of routine clinical care and was not standardized by the study protocol. These variables were used to describe the systemic inflammatory and bone metabolism-related context of the cohort, but they were not considered proof of a mechanistic relationship with TAW status. Patient-level cumulative glucocorticoid exposure was not uniformly documented and therefore could not be included in the formal analyses.

### 2.4. Statistical Analysis

Categorical variables are reported as counts and percentages, and continuous variables as mean ± standard deviation. Between-treatment group comparisons of categorical TAW outcomes were performed using Pearson chi-square tests for three-group comparisons and Fisher exact tests for pairwise comparisons when appropriate. Paired longitudinal comparisons of binary TAW status were assessed separately for the right and left sides using exact McNemar tests based on individual-level transition counts. Proportion confidence intervals were calculated using Wilson 95% confidence intervals, and exact binomial confidence intervals were used for discordant transition rates and progression rates among participants with an existing TAW at baseline. For the primary longitudinal outcome, absolute net percentage-point change and discordant-transition rates with confidence intervals were treated as effect-size measures. For omnibus treatment-group comparisons of binary TAW status, Cramér’s V was calculated. No a priori formal sample-size or power calculation was performed because the follow-up sample size was determined by the number of original participants available for reassessment. To contextualize detectability, a post hoc sensitivity calculation was performed for a simplified one-directional deterioration scenario with no reverse transitions: with *n* = 43, at least 6 deteriorating discordant pairs (14.0 percentage points) are required for a two-sided exact McNemar *p* < 0.05, and a net deterioration rate of approximately 17.8% corresponds to about 80% power under this simplified scenario. Multivariable regression was not attempted because the primary transition outcomes were extremely sparse (no right-sided discordant transitions and only two total left-sided discordances), which would yield unstable and overfitted estimates. Statistical significance was set at *p* < 0.05. Laboratory and subgroup analyses were exploratory, and no formal correction for multiple comparisons was applied; therefore, isolated *p*-values should be interpreted cautiously. Analyses were performed using IBM SPSS Statistics version 22 and verified from the paired transition dataset.

## 3. Results

### 3.1. Cohort Characteristics at Follow-Up

In 2023, the 43 re-evaluated female RA patients had a mean age of 67.79 ± 7.70 years and a mean disease duration of 18.72 ± 6.52 years. Treatment distribution at follow-up was MTX, *n* = 14; IFX, *n* = 14; and TCZ, *n* = 15, and no patient changed treatment subgroup during the follow-up interval. Key cohort characteristics are summarized in Table 1, while the full treatment-group-stratified demographic and treatment-exposure data are provided in Supplementary Table S1. Compared with 2017, the overall sum CRP level decreased significantly, DAS28 decreased significantly from 2.55 ± 0.72 to 2.05 ± 0.66, and serum vitamin D increased significantly from 59.23 ± 27.24 to 99.60 ± 46.82 nmol/L. The key longitudinal laboratory variables are summarized in Table 2; these systemic disease-control data have been previously reported in the broader JCM follow-up article [23]. Detailed inflammatory, lipid, mineral-metabolism and treatment group-stratified laboratory data are provided in Supplementary Table S2.

### 3.2. Temporal Acoustic-Window Status over Time

**Figure 2:**
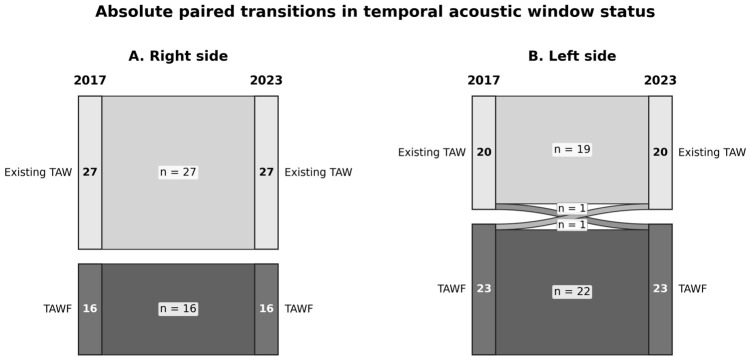
Absolute paired transitions in side-specific TAW status between 2017 and 2023. (**A**) Right side: 27 existing TAW to existing TAW and 16 TAWF to TAWF, with no discordant transitions. (**B**) Left side: 19 existing TAW to existing TAW, 1 existing TAW to TAWF, 1 TAWF to existing TAW, and 22 TAWF to TAWF. TAW: temporal acoustic window; TAWF: temporal acoustic window failure.

At follow-up, right-sided TAW was present in 27/43 patients, while 16/43 exhibited right-sided TAWF. The right-sided distribution was identical at baseline and follow-up; no patient showed a right-sided transition from existing TAW to TAWF or from TAWF to existing TAW. The right-sided TAWF prevalence was 37.2% (95% CI 24.4–52.1%) at both time points, and the exact McNemar test was not statistically significant (*p* = 1.000). On the left side, 20/43 patients had an existing TAW and 23/43 had TAWF at both time points. Two individual-level left-sided discordances were observed: one apparent gain of TAW and one apparent loss. The left-sided TAWF prevalence was 53.5% (95% CI 38.9–67.5%) at both time points, with an exact McNemar *p*-value of 1.000. The discordant transition rate was 0/43 on the right side (0.0%; exact upper 95% bound 8.2%) and 2/43 on the left side (4.7%; exact 95% CI 0.6–15.8%). Among participants with an existing TAW at baseline, the observed progression rate to TAWF was 0/27 on the right side (0.0%; exact 95% CI 0.0–12.8%) and 1/20 on the left side (5.0%; exact 95% CI 0.1–24.9%). Bilateral existing TAW was present in 19/43 patients, unilateral TAW in 9/43, and bilateral TAWF in 15/43 at both baseline and follow-up. The net change in existing TAW was 0.0 percentage points on both sides (Table 3). Absolute side-specific paired transitions are illustrated in Figure 2.

### 3.3. Treatment Subgroup Comparisons

**Figure 3:**
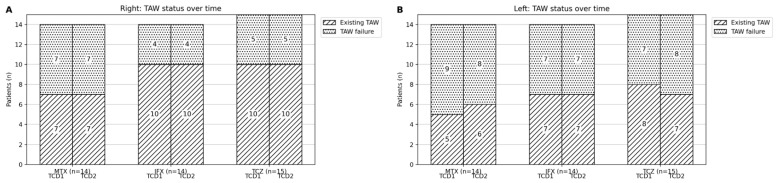
Temporal acoustic window status over time. (**A**) Right-sided TAW status; (**B**) left-sided TAW status. TAW: temporal acoustic window; TAWF: temporal acoustic window failure; TCD1: baseline study; TCD2: follow-up study; MTX: methotrexate; IFX: infliximab; TCZ: tocilizumab.

When stratified by therapy, right-sided existing TAW at baseline was observed in 7/14 MTX, 10/14 IFX, and 10/15 TCZ patients; identical counts were present at follow-up. Left-sided existing TAW was observed in 5/14 MTX, 7/14 IFX, and 8/15 TCZ patients at baseline, and in 6/14 MTX, 7/14 IFX, and 7/15 TCZ patients at follow-up. TAW status did not differ significantly among the MTX, IFX and TCZ groups on either side at either time point. The corresponding omnibus effect sizes were small or negligible (Cramér’s V = 0.188 for the right side at both time points, 0.153 for the left side at baseline, and 0.058 for the left side at follow-up) (Table 4, Figure 3).

The IFX and TCZ groups had numerically higher proportions of existing right-sided TAWs than the MTX group at both time points; however, the corresponding between-group comparisons were not statistically significant. No patient switched between the three treatment subgroups during follow-up, although medication adherence and treatment persistence were not formally assessed.

## 4. Discussion

In this exploratory longitudinal follow-up of female RA patients, the main observation was that binary TAW status showed no detectable net progression over an 8-year interval. This finding is clinically relevant because RA patients may require repeated neurovascular assessment, and an absent transtemporal window can limit MCA insonation, cerebrovascular reserve testing, microembolic monitoring and other TCD/TCCD-based procedures. At the same time, the result must be interpreted cautiously: absence of detectable progression in 43 patients does not prove true biological stability of the temporal bone or exclude small-to-moderate changes in acoustic-window quality. The post hoc detectability analysis further indicates that the present study was primarily capable of identifying relatively large unidirectional deterioration; under the simplified no-reverse-transition scenario, a net deterioration of approximately 17.8% would be required for about 80% power. Therefore, the transition counts, effect-size estimates and confidence intervals are more informative than the non-significant *p*-values alone, and smaller clinically relevant progression remains compatible with the data.

The most direct explanation for TAWF is impaired ultrasound transmission through the temporal bone. Previous studies have linked poor temporal windows to increased temporal bone thickness, altered bone texture and demographic factors, particularly female sex and age. More recent CT- and ultrasound-based studies further suggest that temporal bone thickness and window-quality scores can predict TCCD feasibility [26,27,28,29,30,31,32,33,34]. These data are compatible with our earlier observation that female RA patients had thicker and more heterogeneous temporal bones than controls [35].

RA-related bone remodeling provides a plausible biological context, but not a proven mechanism, for the present findings. Chronic inflammation promotes osteoclast differentiation and activity through cytokine networks and the RANK/RANKL/OPG axis, while also impairing osteoblast-mediated bone formation. These processes contribute to marginal erosions, peri-articular osteopenia and systemic bone loss [8,9,10]. Although the temporal bone is not a typical target of routine RA imaging, our previous findings suggested that cranial bone architecture may also be altered in a way that reduces sonographic permeability [35].

Several explanations may account for the absence of detectable net progression. First, the main structural determinants of TAWF may have already been present before the baseline assessment, with later adulthood characterized by relative classification stability. Second, long-term specialist rheumatological care and improved systemic disease activity may have reduced further inflammatory remodeling. Third, the significant increase in vitamin D levels may indicate a more favorable bone-metabolism context. These explanations remain hypothesis-generating only. The study was not designed to prove that disease control, biological therapy, vitamin D supplementation or any other treatment exposure directly stabilized temporal bone permeability.

Methodological factors are also important. Baseline and follow-up classifications were based on standardized neurosonological examinations performed by the same experienced neurosonographer, and TCCD verification was used only when conventional spectral TCD suggested window failure. This strengthens procedural consistency, but the binary classification may still miss subtle changes in acoustic-window quality. No standardized image archive was available at either time point, precluding retrospective semi-quantitative grading, and formal blinding to the earlier TAW classification was not documented. The two left-sided discordant classifications, one gain and one loss, may reflect true borderline biological change, measurement variability, or small differences related to TCD versus TCCD localization. From a clinical perspective, subtle deterioration below the binary failure threshold could still matter by reducing signal quality, narrowing the usable insonation area, decreasing examination repeatability, or increasing the need for contrast enhancement or alternative imaging. Future longitudinal studies should therefore combine binary feasibility outcomes with prospectively archived images and semi-quantitative window-quality measures.

The treatment subgroup findings should be interpreted with particular caution. No statistically detectable differences in TAW status were observed among MTX, IFX and TCZ groups, but each subgroup contained only 14–15 patients and treatment exposure was not randomized. Although no patient switched between the three treatment subgroups during follow-up, adherence and treatment persistence were not assessed with validated or pharmacy refill-based methods. Therefore, the present data cannot establish treatment equivalence and cannot exclude a treatment-related effect. They only show that no between-group difference was detected in this small exploratory cohort.

### 4.1. Strengths

The main strengths of this study are its rare longitudinal design, paired TAW data, well-characterized RA cohort, and use of the same experienced examiner across time.

### 4.2. Limitations

Limitations include the single-center design, modest sample size, all-female cohort, absence of a healthy follow-up control group, lack of serial CT- or cone-beam CT-based temporal bone thickness and density measurements, lack of semi-quantitative acoustic-window grading, and absence of a standardized neurosonological image archive at either time point. The healthy controls were not prospectively followed because the follow-up study was designed around reassessment of the original RA cohort; therefore, age-related changes cannot be separated from RA-related changes using a contemporaneous longitudinal control group. A detailed statistical comparison of all baseline characteristics between reassessed participants and non-participants was limited by incomplete uniformly available data for the non-participant group, and a formal retained-versus-non-retained analysis of baseline TAW status was not performed. Because the follow-up analysis was restricted to participants who survived and were able to undergo reassessment, the re-evaluated cohort may have preferentially represented healthier survivors. This potential survivor bias may have attenuated the apparent longitudinal change in TAW status and limits the generalizability of the findings. Follow-up examinations were not formally blinded to baseline TAW classification, and formal cohort-specific intra-observer reproducibility data were not available. Cumulative glucocorticoid exposure was not uniformly documented; cumulative steroid dose, current steroid use and duration of use are important confounders for bone metabolism in RA but could not be formally analyzed. Medication adherence and treatment persistence were not assessed using validated adherence instruments or systematic pharmacy-refill metrics. No a priori power calculation was performed, and the very small number of discordant transitions limited the value of multivariable regression and more complex sensitivity analyses. Consequently, absence of statistically detectable progression should not be interpreted as proof of biological stability, and smaller changes remain possible. No formal correction for multiple comparisons was applied, and laboratory or subgroup *p*-values should be interpreted as exploratory.

## 5. Conclusions

In this exploratory 8-year longitudinal study of treated female RA patients, binary TAW status showed no statistically significant longitudinal change. However, the modest sample size and binary classification of acoustic-window status do not exclude subtle changes in temporal bone ultrasound permeability.

TAWF remains clinically relevant because it may limit repeated TCD/TCCD assessments, and baseline documentation of TAW status may help guide the planning of longitudinal neurosonological follow-up.

Larger multicenter studies incorporating direct temporal bone imaging, semi-quantitative acoustic-window grading, and detailed treatment and bone-metabolism data are needed to clarify whether TAWF in RA is fixed, slowly progressive, or potentially modifiable.

## Figures and Tables

**Table 1:** Key cohort characteristics at baseline and follow-up.

Variable	Baseline (2017)	Follow-Up (2023)	Absolute Change	Longitudinal *p*
Age, years	59.44 ± 7.77	67.79 ± 7.70	+8.35 years	—
BMI, kg/m^2^	27.69 ± 5.11	27.58 ± 5.52	−0.11 kg/m^2^	—
Disease duration, years	10.48 ± 6.26	18.72 ± 6.52	+8.24 years	—
Current smokers, n (%)	8 (18.6%)	7 (16.3%)	−2.3 percentage points	1.000

Values are mean ± standard deviation unless otherwise indicated. BMI: Body Mass Index. Full treatment group-stratified demographic, lifestyle, and treatment-exposure data are provided in Supplementary Table S1.

**Table 2:** Key longitudinal inflammatory-, disease activity-, and bone metabolism-related parameters.

Variable	Baseline (2017)	Follow-Up (2023)	Absolute Change	Longitudinal *p*
Sum CRP	6.81 ± 6.09	5.58 ± 8.04	−1.23	0.045
Sum DAS28	2.55 ± 0.72	2.05 ± 0.66	−0.50	<0.001
Osteocalcin	18.66 ± 6.58	19.20 ± 8.65	+0.54	0.835
beta CTx	0.31 ± 0.14	0.35 ± 0.23	+0.04	0.110
Vitamin D, nmol/L	59.23 ± 27.24	99.60 ± 46.82	+40.37 nmol/L	<0.001

Values are mean ± standard deviation. CRP: C-reactive protein; DAS28: Disease Activity Score in 28 Joints; beta CTx: beta-C-terminal telopeptide of type I collagen. Full treatment group-stratified laboratory data are provided in Supplementary Table S2.

**Table 3:** Paired transition matrix of temporal acoustic window status between 2017 and 2023.

Side	Existing TAW -> Existing TAW	Existing TAW -> TAWF	TAWF -> Existing TAW	TAWF -> TAWF	Net Change in Existing TAW	Exact McNemar *p*	Discordant Transition Rate (95% CI)
Right	27	0	0	16	0.0 percentage points	1.000	0/43 (0.0%; 0.0–8.2%)
Left	19	1	1	22	0.0 percentage points	1.000	2/43 (4.7%; 0.6–15.8%)

TAW: temporal acoustic window; TAWF: temporal acoustic window failure; CI: confidence interval. The right-sided exact upper 95% bound refers to the transition rate when no discordant pairs were observed.

**Table 4:** Temporal acoustic window (TAW) status across treatment groups.

	MTX (*n* = 14)	IFX (*n* = 14)	TCZ (*n* = 15)	All Patients (*n* = 43)	*p* MTX vs. IFX vs. TCZ	*p* MTX vs. IFX	*p* MTX vs. TCZ	*p* IFX vs. TCZ
Right TCD1—existing TAW	7	10	10	27	0.467	0.440	0.461	1.000
Right TCD1—TAW failure	7	4	5	16				
Right TCD2—existing TAW	7	10	10	27	0.467	0.440	0.461	1.000
Right TCD2—TAW failure	7	4	5	16				
Left TCD1—existing TAW	5	7	8	20	0.605	0.704	0.462	1.000
Left TCD1—TAW failure	9	7	7	23				
Left TCD2—existing TAW	6	7	7	20	0.931	1.000	1.000	0.700
Left TCD2—TAW failure	8	7	8	23				

Index 1: baseline study; Index 2: follow-up study; MTX: methotrexate; IFX: infliximab; TCZ: tocilizumab; TAW: temporal acoustic window; TCD: transcranial Doppler.

## Data Availability

The data presented in this study, including de-identified patient-level paired TAW transition data underlying the aggregate transition matrices, are available on reasonable request from the corresponding author.

## Supplementary Materials

The following supporting information can be downloaded at: https://www.mdpi.com/article/10.3390/jcm15145654/s1, Table S1. Full treatment-group-stratified demographic, lifestyle and treatment-exposure data; Table S2. Full treatment-group-stratified laboratory data.

## Abbreviations

BHT	Breath-holding test
bDMARD	Biological disease-modifying antirheumatic drug
CRC	Cerebrovascular reserve capacity
CVD	Cerebrovascular disease
DMARD	Disease-modifying antirheumatic drug
PFO	Patent foramen ovale
anti-CCP	Anti-cyclic citrullinated peptide
CRP	C-reactive protein
CT	Computer tomography
DAS28	Disease Activity Score in 28 Joints
ESR	Erythrocyte sedimentation rate
HDL	High-density lipoprotein
IFX	Infliximab
LDL	Low-density lipoprotein
MCA	Middle cerebral artery
MHz	Megahertz
MTX	Methotrexate
OPG	Osteoprotegerin
RA	Rheumatoid arthritis
RANK	Receptor Activator of Nuclear Factor-κB
RANKL	Receptor Activator of Nuclear Factor-κB Ligand
TAW	Temporal acoustic window
TAWF	Temporal acoustic window failure
TCD	Transcranial Doppler
TCCD	Transcranial color Doppler
TCZ	Tocilizumab
