# Supplementary material for: Temporal Acoustic-Window Failure in Female Patients with Rheumatoid Arthritis: An Eight-Year Longitudinal Follow-Up Study
Source: J Clin Med. 2026 Jul 19;15(14):5654. doi: 10.3390/jcm15145654 (PMC13412167; doi:10.3390/jcm15145654)
Supplement: Supplementary file 1 [file jcm-15-05654-s001.zip › jcm-4423156-supplementary.pdf]

Supplementary Tables

Temporal Acoustic Window Failure in Female Patients with Rheumatoid Arthritis: An Eight-Year Longitudinal Follow-Up Study

Table S1. Full treatment-group-stratified demographic, lifestyle and treatment-exposure data.

	MTX (n=14)	IFX (n=14)	TCZ (n=15)	All patients (n=43)	p MTX vs. IFX vs. TCZ	p MTX vs. IFX	p MTX vs. TCZ	p IFX vs TCZ
Age1	60.21±8.69	59.57±7.53	58.60±7.55	59.44±7.77	0.813	0.769	0.621	0.591
Age2	68.79±8.55	68.14±7.49	66.53±7.41	67.79±7.70	0.636	0.874	0.477	0.400
Education in years	13.07±1.94	10.86±3.16	10.73±2.89	11.53±2.86	0.014	0.031	0.007	0.880
BMI1	25.41±5.24	28.74±5.05	28.84±4.64	27.69±5.11	0.065	0.056	0.037	0.914
BMI2	25.42±5.26	28.89±6.39	28.38±4.54	27.58±5.52	0.146	0.125	0.070	0.949
Disease duration1	10.61±7.78	9.21±3.96	11.53±6.62	10.48±6.26	0.648	0.701	0.683	0.310
Disease duration2	19.36±8.40	17.21±3.96	19.53±6.62	18.72±6.52	0.648	0.701	0.780	0.310
Baseline therapy duration in years1	6.89±4.68	6.79±2.78	7.14±4.37	6.94±3.93	0.962	0.804	0.910	0.910
Baseline therapy duration in years2	14.77±9.02	15.50±2.02	13.67±5.51	14.96±6.36	0.576	0.376	0.900	0.448
MTX dosage1	13.75±3.77	13.93±4.78	19.64±2.37	15.77±4.60	<0.001	0.982	<0.001	0.002
MTX dosage2	15.58±4.35	14.38±5.24	18.33±2.89	15.36±4.65	0.456	0.611	0.364	0.295
Biologic treatment duration in years1	-	5.43±1.87	2.93±1.53	4.14±2.100	0.002	-	-	0.001
Biologic treatment duration in years2	-	12.71±2.09	11.87±2.00	11.59±2.95	0.012	-	-	0.354
smoker1 non-smoker1	3 11	1 13	4 11	8 35	0.381	0.596	1.000	0.329
smoker2 non smoker2	3 11	1 13	3 12	7 36	0.527	1.000	1.000	0.598
p				1.000				
alcohol consumption1 non -alcohol consumption1	2 12	3 11	0 15	5 38	0.185	1.000	0.224	0.100
alcohol consumption2 non-alcohol consumption2	1 13	2 12	0 15	3 40	0.320	1.000	0.483	0.224
p				0.500				

Index 1: baseline study; Index 2: follow-up study; BMI: Body Mass Index; MTX: methotrexate; IFX: infliximab; TCZ: tocilizumab.

Table S2. Full treatment-group-stratified laboratory data.

	MTX (n=14)	IFX (n=14)	TCZ (n=15)	All patients (n=43)	p MTX vs. IFX vs. TCZ	p MTX vs. IFX	p MTX vs. TCZ	p IFX vs TCZ
ESR1	17.86±13.40	23.64±15.31	9.13±8.36	16.70±13.72	0.002	0.285	0.008	0.001
ESR2	15.57±11.02	16.29±10.13	12.73±20.67	14.81±14.64	0.030	0.982	0.023	0.023
p				0.408				
Sum ESR1	19.56±13.85	21.89±10.91	12.87±11.82	17.95±12.53	0.021	0.402	0.052	0.008
Sum ESR2	17.86±12.89	21.38±13.96	11.12±12.67	16.63±13.58	0.018	0.720	0.046	0.005
p				0.472				
CRP1	8.91±13.12	4.76±4.97	3.32±5.18	5.61±8.70	0.047	0.352	0.029	0.063
CRP2	6.16±4.99	5.29±11.54	1.72±2.36	4.33±7.39	0.034	0.077	0.012	0,425
p				0.400				
Sum CRP1	7.06±4.11	7.40±8.21	6.05±5.53	6.81±6.09	0.572	0.402	0.363	0.880
Sum CRP2	6.41±4.21	6.99±12.53	3.55±4.61	5.58±8.04	0.108	0.116	0.046	0.747
p				0.045				
DAS28_1	2.61±0.79	2.53±0.62	2.14±0.64	2.42±0.70	0.264	0.874	0.217	0.134
DAS28_2	2.19±0.47	1.95±0.92	1.99±1.19	2.04±0.90	0.056	0.077	0.020	0.813
p				0.003				
Sum DAS28_1	2.79±0.79	2.75±0.50	2.16±0.72	2.55±0.72	0.045	0.943	0.058	0.020
Sum DAS28_2	2.27±0.43	2.01±0.73	1.90±0.75	2.05±0.66	0.064	0.061	0.033	0.652
p				<0.001				
TC1	5.22±0.70	5.54±0.95	5.83±1.34	5.541.05	0.289	0.430	0.118	0.451
TC2	5.47±1.09	5.23±0.88	5.62±1.24	5.45±1.07	0.774	0.720	0.964	0.425
p				0.861				
HDL1	1.46±0.40	1.58±0.37	1.59±0.48	1.55±0.42	0.772	0.462	0.648	0.949
HDL2	1.68±0.25	1.53±0.34	1.76±0.34	1.66±0.32	0.194	0.193	0.719	0.093
p				0.060				
LDL1	2.84±0.72	3.34±0.83	3.60±1.32	3.29±1.04	0.166	0.085	0.126	0.683
LDL2	3.12±1.05	3.05±0.85	3.30±1.05	3.16±0.97	0.831	0.820	0.683	0.591
p				0.909				
TG1	1.27±0.66	1.27±0.66	1.55±0.60	1.37±0.64	0.232	0.720	0.185	0.134
TG2	1.32±0.42	1.65±0.71	1.32±0.48	1.43±0.56	0.308	0.169	0.928	0.217

	MTX (n=14)	IFX (n=14)	TCZ (n=15)	All patients (n=43)	p MTX vs. IFX vs. TCZ	p MTX vs. IFX	p MTX vs. TCZ	p IFX vs TCZ
p				0.406				
Oszteokalcin1	18.09±7.55	19.26±6.82	18.44±5.99	18.66±6.58	0.751	0.508	0.648	0.720
Oszteokalcin2	18.15±9.93	20.66±10.00	18.14±6.14	19.20±8.65	0.791	0.585	0.693	0.685
p				0.835				
beta CTx1	0.34±0.11	0.32±0.13	0.29±0.17	0.31±0.14	0.502	0.752	0.343	0.350
beta CTx2	0.33±0.14	0.40±0.29	0.32±0.22	0.35±0.23	0.758	0.931	0.648	0.488
p				0.110				
D vitamin1	57.16±21.05	65.70±2.00	53.85±26.48	59.23±27.24	0.613	0.585	0.648	0.375
D vitamin2	105.33±38.12	110.68±53.96	83.25±43.26	99.60±46.82	0.182	0.931	0.101	0.141
p				<0.001				
tCalcium1	2.35±0.12	2.35±0.12	2.42±0.25	2.37±0.18	0.935	0.886	0.976	0.685
tCalcium2	2.34±0.13	2.35±0.35	2.36±0.08	2.35±0.22	0.330	0.312	0.648	0.169
p				0.733				
phosphate1	1.12±0.13	1.04±0.11	1.09±0.19	1.08±0.15	0.502	0.259	0.879	0.430
phosphate2	1.11±0.12	1.16±0.40	1.09±0.16	1.13±0.27	0.962	0.752	0.927	0,943
p				0.057				

Index 1: baseline study; Index 2: follow-up study; MTX: methotrexate; IFX: infliximab; TCZ: tocilizumab; ESR: erythrocyte sedimentation rate; CRP: C-reactive protein; DAS28: Disease Activity Score in 28 Joints; TC: total cholesterol; HDL: high-density lipoprotein; LDL: low-density lipoprotein; TG: triglyceride; beta CTx: beta-C-terminal telopeptide of type I collagen.
